# Functional network analysis of p85 and PI3K as potential gene targets and mechanism of oleanolic acid in overcoming breast cancer resistance to tamoxifen

**DOI:** 10.1186/s43141-022-00341-4

**Published:** 2022-04-28

**Authors:** Wilfan Ibadurrahman, Naufa Hanif, Adam Hermawan

**Affiliations:** 1 Laboratory of Macromolecular Engineering, Department of Pharmaceutical Chemistry, Faculty of Pharmacy, Universitas Gadjah Mada Sekip Utara II, Yogyakarta, 55281 Indonesia; 2 Cancer Chemoprevention Research Center, Faculty of Pharmacy, Universitas Gadjah Mada Sekip Utara II, Yogyakarta, 55281 Indonesia

**Keywords:** Oleanolic acid, Breast cancer, Tamoxifen resistance, Bioinformatics

## Abstract

**Background:**

Tamoxifen resistance in estrogen receptor positive (ER+) breast cancer therapy increases, which is the leading cause of cancer treatment failure, as it can impair patients’ prognoses, cause cancer recurrence, metastasis, and death. Combination therapy with compounds is needed to overcome tamoxifen resistance. Oleanolic acid (OA) was known to increase tamoxifen sensitivity in tamoxifen-resistant breast cancer; however, the molecular mechanism of OA and its involvement in overcoming tamoxifen resistance remain unknown and need further investigation. This study was conducted to identify the potential gene targets and molecular mechanisms of OA in overcoming tamoxifen resistance.

**Results:**

A bioinformatic approach for functional network analysis was used in silico by utilizing secondary data in the Gene Expression Omnibus (GEO) database and analyzing them with GEO2R to obtain data on differentially expressed genes (DEGs). The DEG data were further examined with Database for Annotation, Visualization, and Integrated Discovery (DAVID), STRING, cBioPortal website, and Cytoscape with its plugin CytoHubba. Molecular docking was performed to predict the binding properties of OA on the protein encoded by the potential gene. *CD44*, *FGFR2*, *PIK3R1*, and *MDM2* were designated as potential target genes (PTGs), and *PIK3R1* was suspected as the potential gene for OA to overcome tamoxifen resistance. Molecular docking confirms that OA can inhibit p85 activation. *PIK3R1* is suggested to be the potential gene for OA in overcoming tamoxifen resistance in breast cancer therapy.

**Conclusion:**

The predicted molecular mechanism of OA in overcoming tamoxifen resistance involves inhibiting p85 activation, leading to the inhibition of the downstream activity of the PI3K signaling pathway, causing breast cancer to respond to tamoxifen therapy once again. Results of this study need to be validated by further studies, including in vitro and in vivo.

**Supplementary Information:**

The online version contains supplementary material available at 10.1186/s43141-022-00341-4.

## Background

Breast cancer is one of the leading causes of mortality among women worldwide [1]. Over 70% of breast cancers are hormone receptor positive (HR+); that is, they are estrogen receptor positive and progesterone receptor positive, indicating that they are amenable to hormonal therapy such as tamoxifen [2]. Tamoxifen, a selective estrogen receptor modulator (SERM) that can boasts both estrogenic and anti-estrogenic properties depending on the target tissue, is extensively used in early and metastatic stages of breast cancer treatment for HR+ patients, which inhibits estrogen-mediated cell proliferation [3]. The clinical benefits of tamoxifen in HR+ breast cancer therapy are estimated to be between 50 and 60% [4]. Tamoxifen can lower the risk of invasive breast cancer by 30% to 68% compared with placebo [5], by classical mechanism, non-direct DNA binding mechanism, non-genomic mechanism, and dan ligand-independent genomic action [6]. Unfortunately, many breast cancer patients tend to develop tamoxifen resistance during the treatment. Tamoxifen resistance is a major cause of breast cancer treatment failure because it can deteriorate patients’ prognosis, result in cancer recurrence, metastasis, and even death [2, 7].

Previous studies have shown the molecular mechanisms of tamoxifen resistance including alterations in ER signaling, activation of PI3K/AKT/mTOR (PI3K) signaling, and dan activation of NFκB signaling [8]. Tamoxifen resistance also occurs due to cross talk between ER and epidermal growth factor receptor signaling [2], miRNA [9–11]. Epigenetic mechanisms that covers DNA methylation, histone post-translational changes, and chromatin remodeling also regulate breast cancer resistance to tamoxifen [12].

**Fig. 1:**
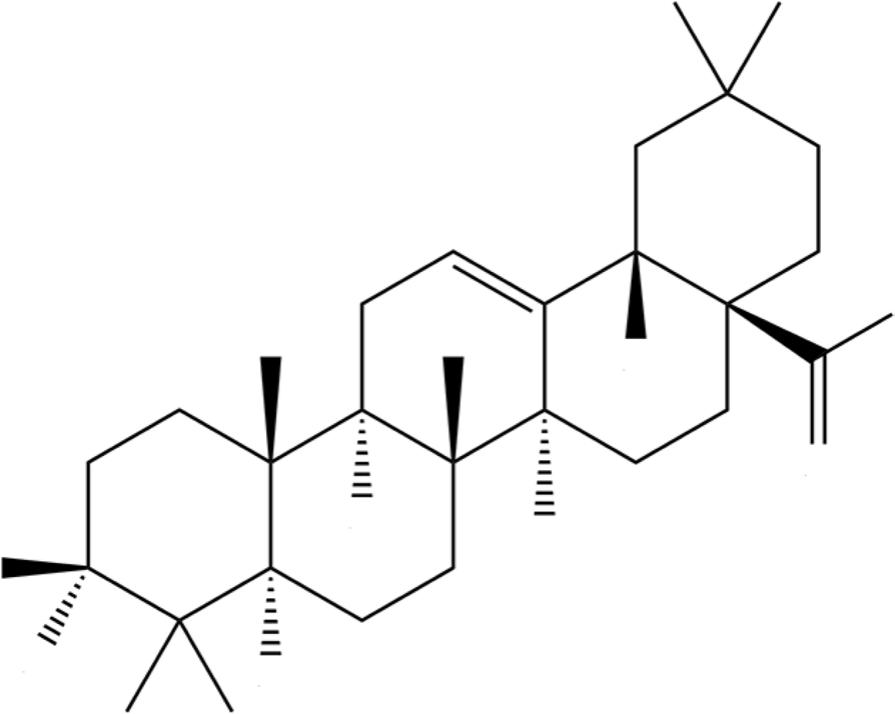
Chemical structure of OA. OA is a pentacyclic triterpenoid compound that has the potential to be used in cancer therapy because it is pharmacologically active

Combinatorial agents can be used to increase the effectiveness of breast cancer treatment [13]. Oleanolic acid (OA, Fig. 1) is a pentacyclic triterpenoid that can be developed as a combination therapy with tamoxifen. It is a bioactive compound particularly abundant in the roots of ginseng and olive trees (*Olea europaea*) [14]. It increases apoptosis in the invasive breast cancer cell line MDA-MB-231 and inhibits the proliferation of the MCF-7 cell line [15, 16]. Gu et al. [16] showed that OA can improve the sensitivity of the tamoxifen-resistant breast cancer cell line MCF-7/TRI. Initially, tamoxifen does not affect MCF-7/TRI. When tamoxifen is administered concurrently with OA, it restores the necessary responses. However, the molecular mechanism of OA and its involvement in overcoming tamoxifen resistance remain unknown and need further investigation.

This study was conducted to identify the potential targets and molecular mechanisms of OA by using a bioinformatic approach of functional network analysis. This approach entails evaluating microarray data using both online and offline software, as has been conducted in a number of prior studies [17–21]. Microarray-based gene expression analysis is a well-established technique for comparing differentially expressed genes (DEGs) in patients suffering from specific diseases [20]. DEGs were generated by analyzing the microarray data obtained from the Gene Expression Omnibus (GEO) data sets. They were subjected to gene ontology (GO) and Kyoto Encyclopedia of Genes and Genomes (KEGG) pathway enrichment analysis to reveal the mechanism by which OA overcomes tamoxifen resistance. The protein–protein interaction (PPI) network and genetic alteration analyses were performed on cBioPortal. Integrating and examining gene data using a bioinformatics approach may help to identify pivotal genes, regulatory pathways, and their roles [18] in treating tamoxifen-resistant breast cancer. This study can provide novel and valuable insights into the potential targets and mechanism of OA and assist the development of therapy in overcoming tamoxifen resistance.

## Methods

### Data collection and processing

The GEO accession number was obtained from a previous research [22] on the use of bioinformatics to identify potential target genes (PTGs) that could be used to overcome tamoxifen resistance. The GEO accession number codes are GSE67916 and GSE85871. The microarray data of tamoxifen-resistant MCF-7 cells were collected from GSE67916 [23], which contained eight and two samples of tamoxifen-sensitive and tamoxifen-resistant MCF-7 cells, respectively. The mRNA microarray data of MCF-7 cells treated with OA were obtained from the public database GSE85871 [24], which included two samples of MCF-7 cells treated with 10 μM OA and DMSO as solvent control for 24 h. GEO2R, an online program for GEO data set analysis based on the R programming language, was used to process the data. The GEO2R utilized methods such as the Benjamini and Hochberg (false discovery rate) and *t-*test to calculate the FDR and p-value in order to determine the DEGs on both GSE67916 and GSE85871 [19, 20].

The distribution of data for GSE67916 and GSE86871 was quite good (Supplementary Figure 1). Relevant DEGs were chosen on the basis of *p* < 0.05, a | log (fold change) | > 1, and the presence of a gene symbol. Upregulated DEGs were considered if the log2 (fold change) > 1 and log2 (fold change) < − 1 for downregulated DEGs [25]. The potential OA target genes against tamoxifen-resistant breast cancer were curated by generating a Venn diagram from GSE74391 and GSE85871 by using Venny 2.0 [26]. The Venn diagram’s overlap called the potential oleanolic acid target gene (OTG) was further analyzed.

### Gene ontology and KEGG pathway enrichment analysis

GO and KEGG pathway enrichment analyses were used to investigate the biological function and molecular mechanism of DEGs. The Database for Annotation, Visualization, and Integrated Discovery (DAVID) v6.8 [27, 28] was used to analyze the OTGs and obtain GO and KEGG pathway enrichment. GO analyses were conducted in accordance with three criteria: biological process, cellular component, and molecular function. Fisher’s exact test was used to measure gene enrichment in annotation terms. When the entire family of tests is considered, the smallest significance level at which the given hypothesis would be rejected is known as the *p* value*. p* < 0.05 was chosen as the cutoff value.

### Analysis of the PPI network and selection of hub genes

OTGs were also used to analyze the PPI network. It was constructed using STRING-DB v11.0 [29] with a medium confidence score of > 0.4 and visualized using Cytoscape [30]. The CytoHubba plugin was utilized to examine genes with a degree of > 5. The top 20 genes were chosen as hub genes based on their highest degree as determined by the CytoHubba plugin under default settings [31].

### Analysis of the genetic alterations of PTGs

The genetic alterations analysis of PTGs were assessed via cBioPortal using samples from 18 breast cancer studies [32, 33] as query genes. Genome change visualization and pathway alterations were examined via OncoPrint on the breast cancer study with the highest genetic alterations.

### Molecular docking

The binding properties of OA on the p85 regulatory subunit were predicted by molecular docking. Computational prediction was performed on a Windows 10 computer equipped with an Intel Core™ i5 10th Generation processor and 8 GB of RAM. MOE 2010 (licensed from the Faculty of Pharmacy Universitas Gadjah Mada) was used to simulate docking, calculate the RMSD docking score, and visualize interactions. According to rcsb.org, the PDB ID for p85 was 4L2Y. The OA structure was drawn by copying Canonical Smiles on PubChem. Afterward, the structure was subjected to conformational search and energy minimization in MOE via the Energy Minimize Menu. For docking simulation, London dG was used for Rescoring 1 and Rescoring 2. Triangle Matcher was utilized for the score function and placement setting, and Forcefield was applied to refine the docking result from 30 retain settings. The results of this method were used to determine the conformation of the ligand with the lowest binding interaction with its receptor.

## Results

### Data collection and processing

**Fig. 2:**
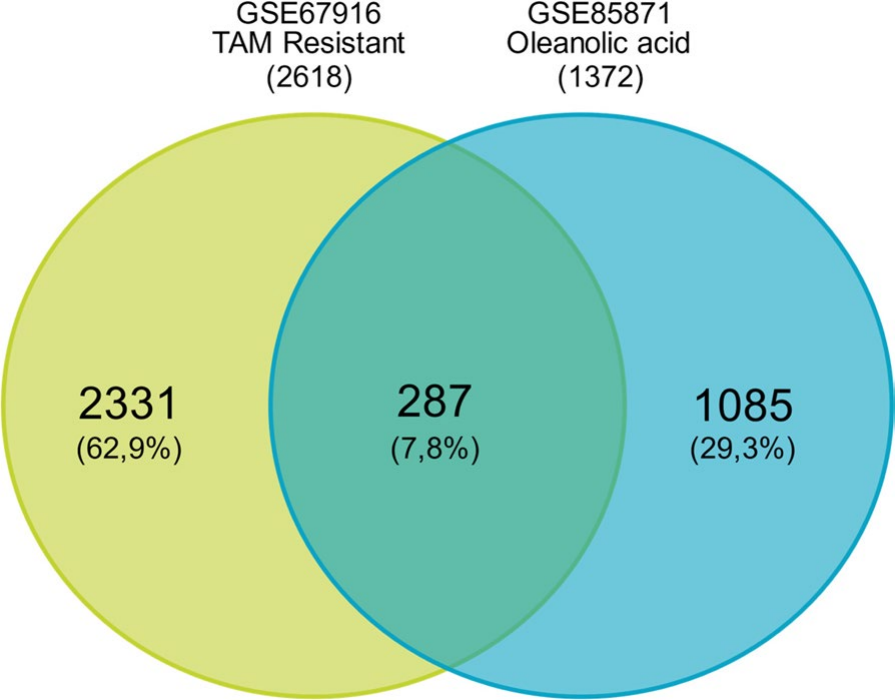
Venn diagram of tamoxifen-resistant and OA-treated MCF-7 breast cancer cells. The Venn diagram’s overlap represents a group of genes expressed by MCF-7 during tamoxifen resistance and OA treatment

This study was performed to identify the PTGs and mechanisms of OA in overcoming tamoxifen resistance in breast cancer. The data were a collection of microarray data (data series) from the tamoxifen-resistant MCF-7 cell line (GSE67916) and the MCF-7 cell line treated with OA (GSE85871). Furthermore, these data have never been used to identify the molecular targets and mechanisms of action of OA in the treatment of tamoxifen resistance in breast cancer. A total of 3388 DEGs were obtained from the results of the sorting and analysis of GSE67916 by using GEO2R (Supplementary Table 1). These DEGs included 976 upregulated genes and 2412 downregulated genes. Moreover, 1477 DEGs were identified from the GSE85871 data series, consisting of 809 upregulated and 668 downregulated genes (Supplementary Table 2). According to the overlap of the Venn diagrams, 287 OTGs exist (Fig. 2). OTGs are genes that are overexpressed or underexpressed in tamoxifen-resistant events or in response to OA treatment.

### Gene ontology and KEGG pathway enrichment analysis

Functional annotation analyses, such as GO and KEGG pathway analysis, aim to elucidate the biological function and molecular mechanism of OTGs. According to the results of GO and KEGG pathway enrichment analysis, 287 genes are involved in 66 biological processes (Supplementary Table 3), 33 cellular components (Supplementary Table 4), 16 molecular functions (Supplementary Table 5), and 19 signaling pathways (Supplementary Table 6). The results of the top GO are summarized in Table 1. Several cellular signaling pathways are frequently involved in breast cancer signaling processes (Table 2).

**Table 1:** Top gene ontology (GO) of OTGs. It is sorted on the basis of the smallest *p* value

Term	*p* value	Genes
Biological process		
GO:0060337~type I interferon signaling pathway	5.29453E-07	*SP100*, *IFI27*, *OAS1*, *OAS2*, *MX1*, *HLA-B*, *IFI6*, *HLA-C*, *IFIT1*, *HLA-G*
GO:0098609~cell-cell adhesion	1.94184E-05	*SH3GLB2*, *LAD1*, *HSP90AB1*, *TACSTD2*, *CAPG*, *ENO1*, *RANGAP1*, *TMEM47*, *EIF2S3*, *EEF1D*, *EPB41L1*, *MYO6*, *TAGLN2*, *SPTAN1*, *SPTBN1*, *PICALM*
GO:0000122~negative regulation of transcription from RNA polymerase II promoter	0.000299991	*SP100*, *RARG*, *IKZF1*, *PSEN1*, *RELA*, *SOX2*, *TPR*, *TBL1X*, *TAF9B*, *TFAP2A*, *TFAP2C*, *JUND*, *MSX2*, *H2AFY*, *DICER1*, *VEGFA*, *EID1*, *RBL1*, *BCL6*, *IFI27*, *NFIB*, *MDM2*, *TCF3*, *TRIM33*, *FGFR2*
Cellular component		
GO:0005737~cytoplasm	1.63681E-08	*IKZF1*, *ENO1*, *IFIT1*, *ACTB*, *SOX2*, *CDH1*, *NEK2*, *GLUL*, *MAP3K7*, *TNS3*, *TPD52*, *SH3GLB2*, *ACOT7*, *CDKL5*, *MBNL2*, *ADAM10*, *UBE4B*, *PPP4R3A*, *DICER1*, *ACLY*, *EID1*, *ARC*, *PRKAR1A*, *TRIM14*, *OBSL1*, *SKAP2*, *SET*, *MAX*, *STC1*, *GMPR*, *PIK3R1*, *DDX60*, *LPP*, *ZFP36L2*, *EPB41L1*, *EPB41L2*, *TPR*, *PSAP*, *MYO6*, *G3BP1*, *SOCS6*, *SPTBN1*, *SCAI*, *TFAP2A*, *PRNP*, *DTNA*, *GADD45B*, *CEP350*, *IFI44*, *EIF1*, *GULP1*, *EIF2S3*, *CAPRIN1*, *CDK2*, *MDM2*, *GNAS*, *NMT1*, *TCF3*, *FARSA*, *CDK13*, *FGFR2*, *FERMT2*, *RET*, *DAZAP2*, *ZCCHC11*, *RBM25*, *HSP90AB1*, *CITED1*, *LYST*, *BICD2*, *DUSP10*, *MECOM*, *GPER1*, *POLH*, *ZNF365*, *BTN3A3*, *KIF22*, *RANGAP1*, *ZDHHC17*, *DDX19A*, *OAS1*, *DNAJC7*, *SLC7A8*, *OAS2*, *HNRNPH1*, *EEF1D*, *TFF3*, *RAPGEF2*, *GART*, *MAPRE2*, *CD44*, *NFAT5*, *SP100*, *SRSF1*, *CAPG*, *TYMS*, *TANK*, *RELA*, *HSPD1*, *SH3BGRL*, *CCL5*, *SRSF10*, *PDLIM7*, *ARFGEF1*, *SPAG9*, *GSN*, *PLEKHA1*, *MX1*, *RANBP9*, *PTPN11*, *PSMB9*, *VEGFA*, *KITLG*, *XPNPEP1*, *CCNG2*, *ALMS1*, *FAS*, *MBTPS2*
GO:0070062~extracellular exosome	6.52789E-07	*SERPINA3*, *LAD1*, *ZCCHC11*, *PATJ*, *SERPINA1*, *HSP90AB1*, *ITGB5*, *ABAT*, *ENO1*, *ACTB*, *EFEMP1*, *GM2A*, *CDH1*, *SMPD1*, *THSD7A*, *SPTAN1*, *GLUL*, *ACOT7*, *H2AFY*, *ATP6AP2*, *HLA-B*, *HPCAL1*, *HLA-C*, *ADAM10*, *RNASE4*, *ACLY*, *CLDN3*, *SLC7A8*, *DNAJC7*, *TFF3*, *B3GNT2*, *TAGLN2*, *DNASE2*, *ATP6V0D1*, *IL6ST*, *GART*, *S100A8*, *CD44*, *CFB*, *ATP6V1A*, *GRN*, *RALB*, *SRSF1*, *TACSTD2*, *CAPG*, *SLC1A4*, *HSPD1*, *SH3BGRL*, *EPB41L2*, *PSAP*, *MYO6*, *TSPAN3*, *SPTBN1*, *SPAG9*, *PRNP*, *SLC16A1*, *GSN*, *PLEKHA1*, *F12*, *LAMB1*, *RAB27B*, *PSMB9*, *GATM*, *EIF2S3*, *XPNPEP1*, *XPNPEP3*, *FAT1*, *GNAS*, *NUCB1*, *FAS*, *P4HB*, *CD22*
GO:0016020~membrane	2.55671E-06	*RET*, *NRP1*, *HSP90AB1*, *IL1RAP*, *ENO1*, *ACTB*, *TM7SF2*, *FADS3*, *LAPTM4B*, *CDH1*, *SPTAN1*, *HLA-B*, *HPCAL1*, *DIO2*, *HLA-C*, *ADAM10*, *BTN3A3*, *SDHC*, *HLA-G*, *DDX19A*, *ACLY*, *SLC7A8*, *DNAJC7*, *PRKAR1A*, *OAS2*, *HNRNPH1*, *RAPGEF2*, *ATP6V0D1*, *ERGIC3*, *SEC22B*, *IL6ST*, *TACSTD2*, *RRBP1*, *SLC1A4*, *PSEN1*, *PIK3R1*, *HSPD1*, *AKAP1*, *ABR*, *UGCG*, *MYO6*, *GCNT2*, *PSMF1*, *SLC16A1*, *CEP350*, *C1GALT1*, *VEGFA*, *KITLG*, *G6PC3*, *PDE10A*, *GGCX*, *CAPRIN1*, *FAT1*, *GNAS*, *NUCB1*, *FAS*, *FARSA*, *FGFR2*, *PICALM*
Molecular function		
GO:0005515~protein binding	9.49741E-11	*PATJ*, *TRIO*, *TRRAP*, *IKZF1*, *ENO1*, *IFIT1*, *ACTB*, *SOX2*, *LAPTM4B*, *EFEMP1*, *TDO2*, *CDH1*, *NEK2*, *CCNL2*, *GLUL*, *MAP3K7*, *TNS3*, *TPD52*, *SH3GLB2*, *WSB1*, *ACOT7*, *MSX2*, *TPM1*, *HPCAL1*, *ADAM10*, *PRPF4B*, *DICER1*, *CDC25C*, *PRLR*, *ACLY*, *EID1*, *PRKAR1A*, *TAGLN2*, *RPP25*, *ATP6V0D1*, *IL6ST*, *OBSL1*, *S100A8*, *SKAP2*, *SET*, *MAX*, *TACSTD2*, *DAPP1*, *PSEN1*, *TRMT2B*, *PIK3R1*, *DDX60*, *LPP*, *ZFP36L2*, *STIP1*, *EPB41L1*, *TPR*, *PSAP*, *MYO6*, *G3BP1*, *PSMF1*, *SOCS6*, *SPTBN1*, *SCAI*, *TFAP2A*, *PLK4*, *PRNP*, *TFAP2C*, *DTNA*, *JUND*, *GADD45B*, *CEP350*, *ASPSCR1*, *SREK1IP1*, *RAB27B*, *FOXN3*, *PHC3*, *EIF2S3*, *BCL6*, *IL7*, *FUBP1*, *CDK2*, *MDM2*, *FAT1*, *GNAS*, *TCF3*, *FARSA*, *CDK13*, *CD22*, *FGFR2*, *FERMT2*, *PICALM*, *RET*, *SERPINA3*, *DAZAP2*, *NRP1*, *ZCCHC11*, *RARG*, *RBM25*, *SERPINA1*, *HSP90AB1*, *CITED1*, *ITGB5*, *CHD7*, *LYST*, *BICD2*, *WDR43*, *SPTLC1*, *MECOM*, *ZC3H7B*, *GPER1*, *SMPD1*, *C9ORF16*, *SPIN1*, *SPTAN1*, *POLH*, *RBM5*, *ZNF365*, *MSMB*, *SS18*, *HELLS*, *H2AFY*, *SECISBP2L*, *ATRX*, *ATP6AP2*, *KIF22*, *RANGAP1*, *ZDHHC17*, *DDX19A*, *SIKE1*, *RBL1*, *OAS1*, *DNAJC7*, *SLC7A8*, *OAS2*, *HNRNPH1*, *EEF1D*, *TFF3*, *RAPGEF2*, *SEC22B*, *MAPRE2*, *CD44*, *VAMP3*, *NFAT5*, *SP100*, *GRN*, *RALB*, *SRSF1*, *IFI6*, *CAPG*, *PBXIP1*, *TANK*, *RELA*, *HSPD1*, *AKAP1*, *RXRB*, *ERBB4*, *CCL5*, *TBL1X*, *TAF9B*, *SRSF10*, *PDLIM7*, *KCNJ3*, *MPZL2*, *ARFGEF1*, *SPAG9*, *GSN*, *PLEKHA1*, *F12*, *C1GALT1*, *MX1*, *SAMD4A*, *RANBP9*, *PTPN11*, *KLF3*, *PSMB9*, *VEGFA*, *KLF6*, *KITLG*, *ALMS1*, *NUCB1*, *FAS*, *P4HB*, *CYB561*, *TRIM33*
GO:0098641~cadherin binding involved in cell-cell adhesion	0.000107912	*SH3GLB2*, *LAD1*, *HSP90AB1*, *CAPG*, *ENO1*, *RANGAP1*, *EIF2S3*, *CDH1*, *EEF1D*, *EPB41L1*, *MYO6*, *TAGLN2*, *SPTAN1*, *SPTBN1*, *PICALM*
GO:0042803~protein homodimerization activity	0.000523424	*TFAP2A*, *TPD52*, *SP100*, *ACOT7*, *SLC16A1*, *CITED1*, *MAX*, *ADAM10*, *ABAT*, *TYMS*, *PRLR*, *HLA-G*, *RELA*, *VEGFA*, *MECOM*, *XPNPEP1*, *ERBB4*, *CCL5*, *TPR*, *TCF3*, *PSMF1*, *IL6ST*, *FGFR2*, *ZNF365*

**Table 2.** KEGG pathway of OTGs. The signaling pathways in the table are usually involved in cancer

Term	*p*-Value	Genes
hsa05200:Pathways in cancer	0.000882275	*RET*, *RALB*, *HSP90AB1*, *MAX*, *PTGER3*, *LAMB1*, *PIK3R1*, *RELA*, *VEGFA*, *RXRB*, *KITLG*, *MECOM*, *CDH1*, *TPR*, *CDK2*, *MDM2*, *GNAS*, *FAS*, *FGFR2*
hsa04010:MAPK signaling pathway	0.031747523	*DUSP10*, *JUND*, *GADD45B*, *MECOM*, *MAX*, *FAS*, *RAPGEF2*, *CACNA1D*, *MAP3K7*, *RELA*, *FGFR2*
hsa04151:PI3K-Akt signaling pathway	0.045763549	*HSP90AB1*, *ITGB5*, *LAMB1*, *PIK3R1*, *PRLR*, *RELA*, *VEGFA*, *KITLG*, *G6PC3*, *IL7*, *CDK2*, *MDM2*, *FGFR2*
hsa04115:p53 signaling pathway	0.046007991	*GADD45B*, *CCNG2*, *CDK2*, *MDM2*, *FAS*
hsa05205:Proteoglycans in cancer	0.049149374	*ITGB5*, *ERBB4*, *MDM2*, *FAS*, *PTPN11*, *PIK3R1*, *CD44*, *ACTB*, *VEGFA*

### Analysis of the PPI network and selection of hub genes

**Fig. 3:**
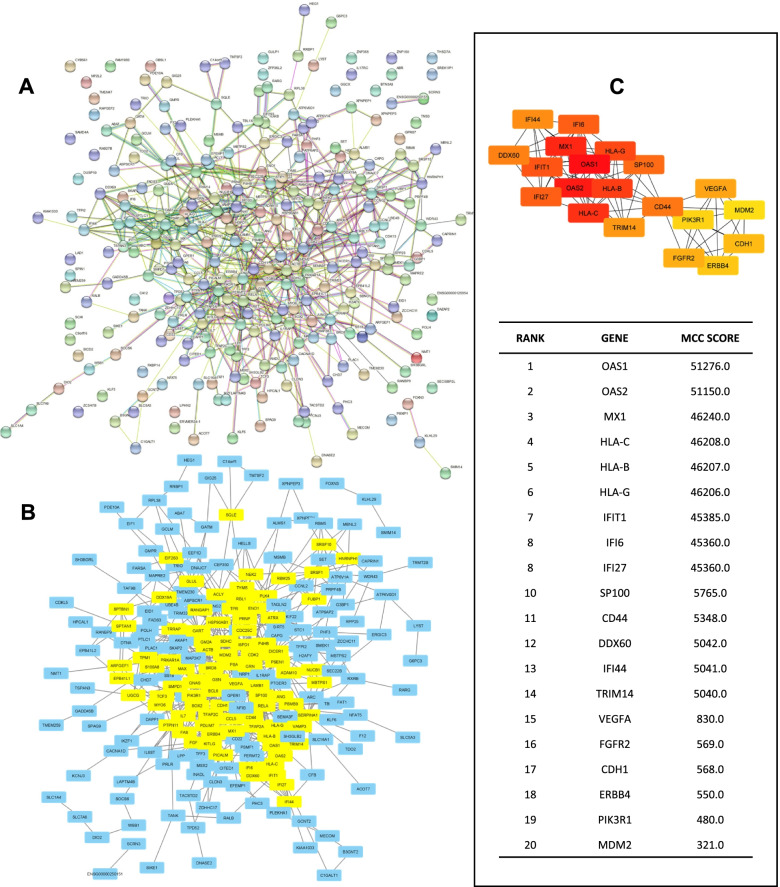
Interactions between proteins encoded by OTGs. **A** STRING-DB analysis of the PPI network of proteins involved in overcoming tamoxifen resistance by OA. **B** The PPI filtered results subjected to Cytoscape 3.8.0 to filter out proteins with a value of 5° or more, which are shown in yellow. **C** Top 20 hub genes with the highest MCC score analyzed using the CytoHubba plugin

PPI analysis was conducted to determine the interactions among the 287 OTGs. The results showed 269 genes encoding proteins in *Homo sapiens* (humans). In Fig. 3A, the proteins encoded by these genes were represented as nodes, and the relationships between them were referred to as edges. Additionally, 269 nodes and 653 edges, along with a PPI enrichment value of 4.55e-15 and an average local clustering coefficient of 0.372, were visible. A low *p* value of PPI enrichment indicated that the nodes were not random, and the observed edge count was significant. The clustering coefficient expressed the degree to which the nodes in a network were connected. Networks with a high degree of connectivity had a high value. After the interaction data were obtained, they were exported to Cytoscape 3.8.0, whose primary goal is to identify the most interacting proteins. The findings are depicted in Fig. 3B, and yellow indicates a protein with a degree value of ≥ 5.

The CytoHubba plugin of Cytoscape is used to define a hub gene. This plugin sorts up to 20 genes according to their maximal clique centrality (MCC) score. Then, as illustrated in Fig. 3C, the 20 genes with the highest scores are considered hub genes. In this study, PTGs were chosen on the basis of hub genes and their involvement in the breast cancer signaling pathway (Table 2). The genes designated as PTGs were *CD44*, *FGFR2*, *PIK3R1*, and *MDM2*. *CD44* encodes a nonkinase transmembrane glycoprotein receptor that promotes the proliferation, survival, and migration of cancer cells [34]. *FGFR2* encodes a receptor tyrosine kinase for fibroblast growth factors, which are frequently implicated in tumor growth [35]. *PIK3R1* encodes the p85α regulatory subunit of the PI3K enzyme, which is involved in cancer growth [2]. *MDM2* encodes the MDM2 protein, which acts as a major negative regulator of the tumor suppressor p53 [36]. p53 is a barrier to cancer cell growth [37], and it is a druggable target, along with ERα [38]. These PTGs are potential targets for OA in overcoming tamoxifen resistance in breast cancer. However, the most dominant target gene remains unknown.

### Analysis of genetic alterations of PTGs

**Fig. 4:**
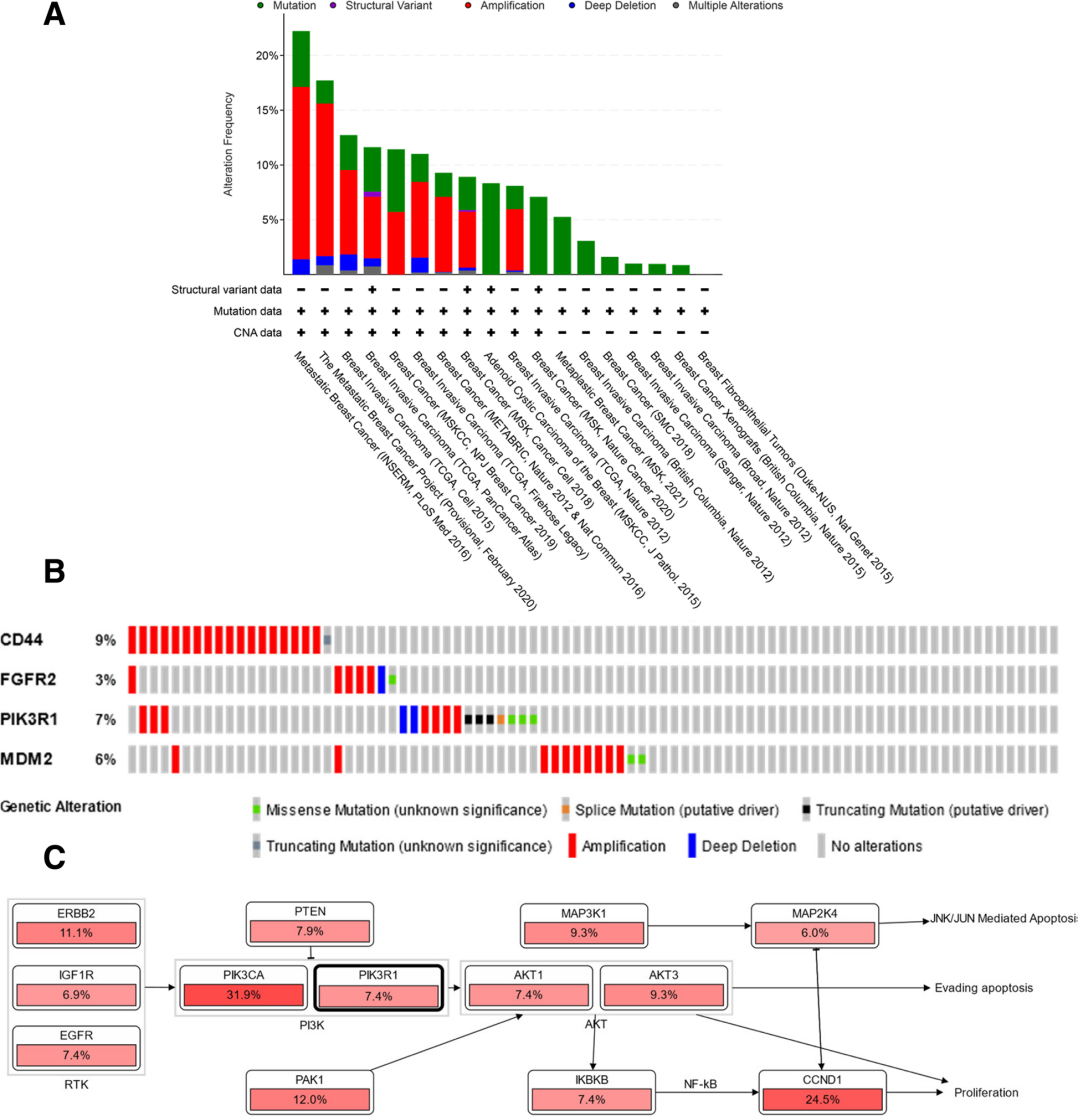
Analysis of genetic alterations and pathway in the cancer of PTG from breast cancer studies via cBioPortal. **A** A summary of changes in *CD44*, *FGFR2*, *PIK3R1*, and *MDM2* across 18 studies on breast cancer. **B** Summary of genetic alterations in *CD44*, *FGFR2*, *PIK3R1*, and *MDM2* in breast cancer samples based on the study of Lefebvre et al. (2016). **C** Pathways related to genetic alterations in PIK3R1 on the PI3K/AKT signaling pathway

This analysis aims to evaluate the genetic profile of PTGs across multiple breast cancer studies because the genes associated with cancer are frequently altered genetically. In Fig. 4A, the cBioPortal database contains 18 breast cancer studies. The graphs show the percentage of patients with genetic alterations, which include mutations, amplifications, deep deletions, and multiple alterations to PTGs that have been entered into the cBioPortal. Green, red, blue, and gray denote mutation, amplification, deep deletion, and multiple alterations, respectively. However, a study conducted by the BRCA *Institut National de la Santé et de la Recherche Médicale* (INSERM) 2016 [39] was chosen for further analysis. This study was chosen because it included a higher proportion of patients with genetic alterations than that of patients in other studies on breast cancer. Additionally, this study included patients who had metastatic breast cancer and those who were resistant to chemotherapy. The majority (66.2%) of the sample had HR+ breast cancer.

The OncoPrint section illustrated the genetic alteration of each target gene. The results were quite diverse, i.e., *CD44* (9%), *FGFR2* (3%), *PIK3R1* (7%), and *MDM2* (6%). The percentage of genetic variants is depicted in Fig. 4B. The majority of these genetic changes occurred as amplification (19.91%) and mutations (5.09%). The pathway section contained an additional analysis of the signaling pathways involving these genes. The results indicated that the RTK-RAS-PI(3) K (PI3K/AKT) and TP53 pathways are critical for cancer cell control, as illustrated in Fig. 4C. Additionally, *PIK3R1*, which is involved in the PI3K-AKT pathway, and *MDM2*, which is implicated in the TP53 pathway, are potential targets that play a critical role in the treatment of OA. However, the involvement of *PIK3R1* in the PI3K/AKT pathway in breast cancer should be further investigated because *PIK3R1* is a gene encoding a subunit of the PI3K protein. The PI3K protein has an effect on various signaling pathways, including the one that involves the MDM2 protein; the P13K protein also participates in the tamoxifen resistance mechanism [40]. As a result, *PIK3R1* was identified as a potential gene for OA to overcome tamoxifen resistance.

### Molecular docking

According to the results of the cBioPortal and KEGG pathway enrichment analysis, *PIK3R1*, a gene encoding the protein p85, is one of the genes that undergoes many alterations and contributes to cancer signaling pathways. Moreover, the protein encoded by this gene plays an important role in PI3K/AKT, an essential pathway in breast cancer signaling [40]. Numerous studies [40–42] also mention that p85 is involved in the process of resistance in breast cancer cells. As a result, molecular docking is required to observe the interaction of OA with p85.

**Fig. 5:**
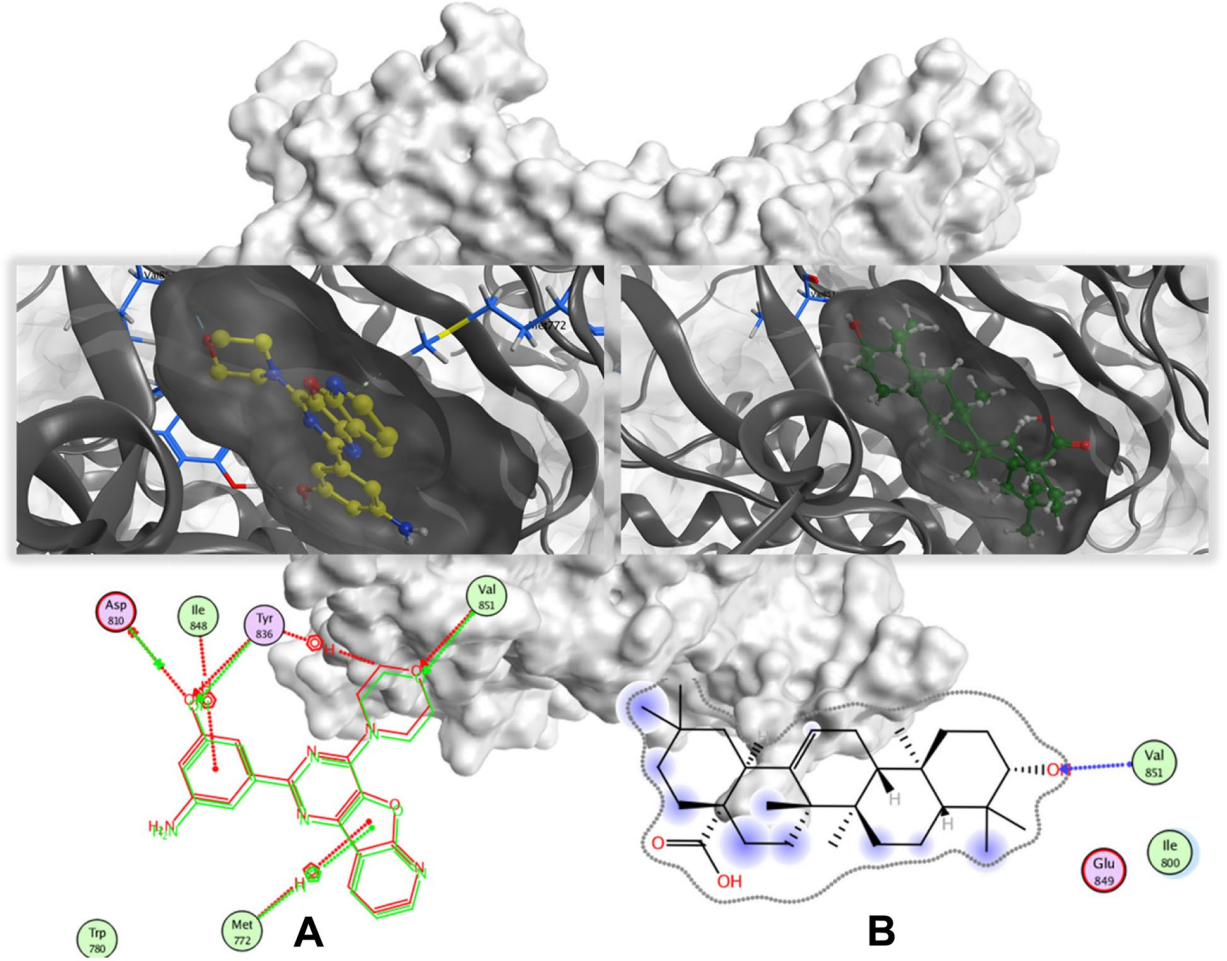
Visualization of ligand interaction of (**A**) native ligand, XXK (3-amino-5-[4-(morpholin-4-yl)pyrido[3′,2′:4,5]furo[3,2-d]pyrimidin-2-yl]phenol) and (**B**) OA with p85 (PDB ID: 4L2Y) by using MOE

The native ligands of each protein consist of p85 complexes XXK (3-amino-5-[4-(morpholine-4-yl)pyrido[3′,2′:4,5]furo[3,2-d]pyrimidin-2-yl]phenol). The docking score of the interaction of OA with p85 was higher than that of its native ligands, indicating a lower binding affinity to each receptor. This result is consistent with the involved amino acid on OA for each receptor. The native ligand of p85 has three amino acids, which interacted through Arene-H (Met772) and side chain donor (Tyr836 and Val851), as seen in Table 3 and Fig. 5A. The docking score of OA was higher because only one amino acid, Val851, interacted on p85 via a backbone donor bond, as illustrated in Table 3 and Fig. 5B.

**Table 3:** Molecular docking summary. The docking score of oleanolic acid (OA) is higher than that of its native ligands

Native ligand							Oleanolic acid (OA)					
PDB ID	Docking score	RMSD (Å)	Ligand atom	Amino acid	Binding type	Distance	Docking score	RMSD (Å)	Ligand atom	Amino acid	Binding type	Distance
P85 (4L2Y)			C	Met772	ArH	3,93						
	− 14.31	0.383	O	Tyr836	ScD	2.00	− 13.52	2.038	O	Val851	BbD	2.12
			O	Val851	ScD	1.85

OA can alleviate the symptom of renal ischemia–reperfusion injury through the PI3K/AKT signaling pathway [43]. OA and its derivatives contain α,β-unsaturated carbonyl moiety and various amide substituents that inhibit the PI3K/AKT/mTOR pathway [44]. OA induces autophagic cell death via PI3K/AKT1/mTOR and ROS-dependent pathways [43]. PI3K is a heterodimer composed of a p110 catalytic subunit and a p85 regulatory subunit [45]. Although the docking score of OA was slightly lower than that of the native ligand (Table 3), Val851 binds to both ligands, as shown in Fig. 5A and B. These results indicated that OA can substitute for native ligands because of its similar amino acid binding (Val851) even though it lacks the strength to compete with native ligands because of its docking score.

## Discussion

**Fig. 6:**
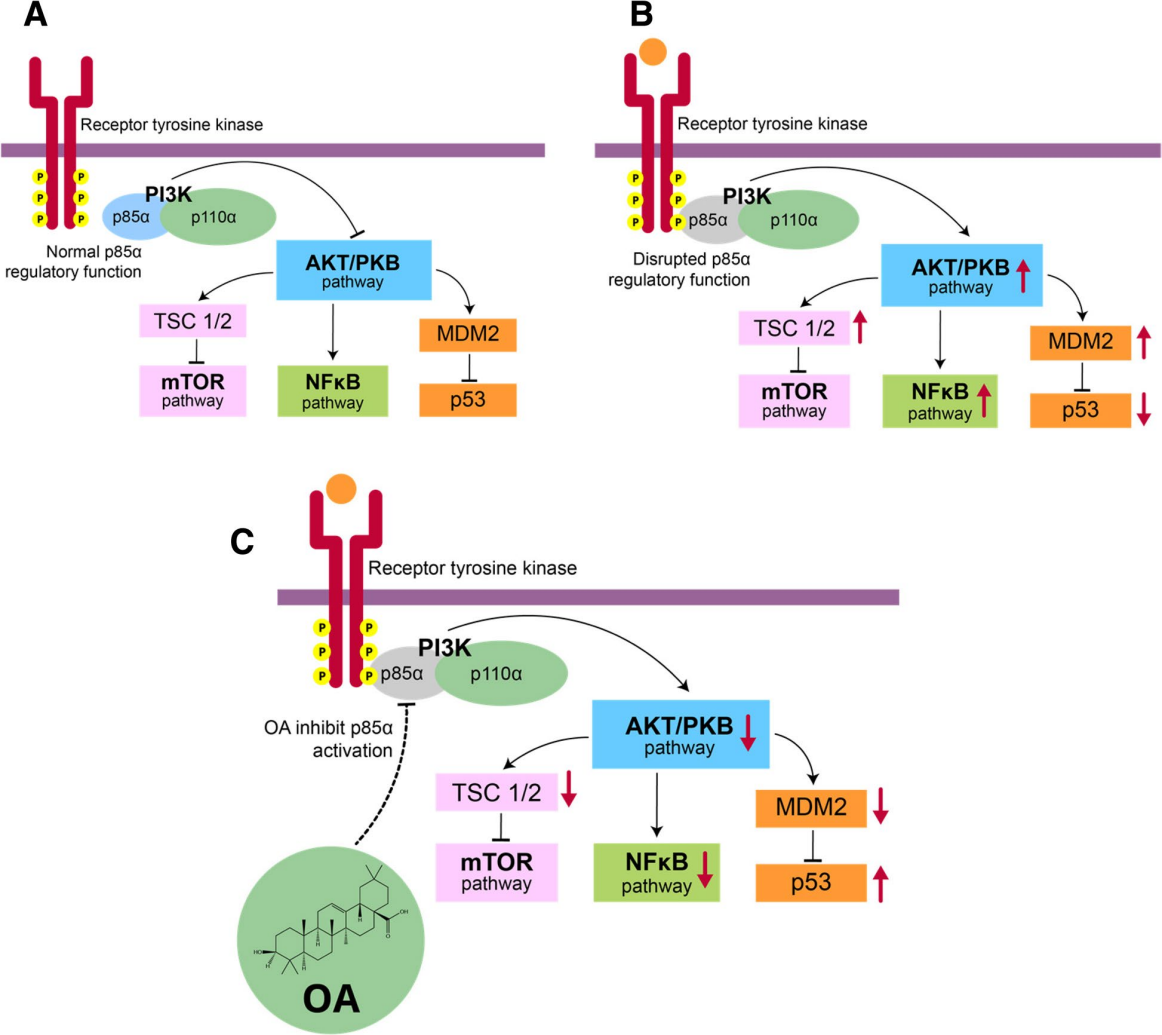
Proposed molecular mechanism of OA in overcoming tamoxifen resistance. **A** The regulatory function of p85α is normal. **B** The regulatory function of p85α is disrupted. **C** OA reinstates the regulatory function of p85α subunit

Through functional network analysis, a candidate gene, namely *PIK3R1*, which encodes the p85α regulatory subunit of PI3K [2], was identified for targeted therapy. This finding was confirmed by the KEGG pathway analysis through which the involvement of PI3K/AKT signaling pathway was identified. Results of this study is supported by a previous study that suggested PIK3 inhibitors to sensitize luminal A breast cancer cells to tamoxifen [46]. A clinical trial of PI3K inhibitor like buparlisib and alpelisib in combination with Tamoxifen was performed in premenopausal patients with HR+/HER2–locally advanced or metastatic breast cancer [47]. Those findings highlighted the importance of PI3K inhibitor in enhancing tamoxifen efficacy. Phosphatidylinositol 3-kinases (PI3Ks) constitute a family of intracellular heterodimeric lipid kinase enzymes that respond to signals from G protein-coupled receptors and receptor tyrosine kinases (RTKs), such as HER, FGFR, and IGF-1. The PI3K enzyme is a heterodimer composed of two subunits: the p110 catalytic subunit and the p85 regulatory subunit [2, 48]. This enzyme regulates cell growth, proliferation, survival, differentiation, metabolism, migration, genome stability, protein synthesis, and angiogenesis [2]. Figure 6 illustrates the proposed chart that explains the role of OA in overcoming breast cancer resistance to tamoxifen.

When activated by RTK, PI3K converts phosphatidylinositol 4,5-bisphosphate (PIP2) to phosphatidylinositol 3,4,5-triphosphate (PIP3). PIP3 accumulates in the plasma membrane and attracts plasma membrane-localized phosphoinositide-dependent kinase 1 (PDK1) and protein kinase B/AKT, a critical mediator of the PI3K signaling pathway. AKT is phosphorylated by PDK1 and PDK2 and becomes active, thereby stimulating downstream pathways such as the mammalian target of rapamycin (mTOR) via tuberous sclerosis complex 1/2 (TSC1/2), NFκB, and MDM2 among others. The tumor suppressor protein phosphatase and tensin (PTEN) homolog catalyzes the dephosphorylation of PIP3 to PIP2, so it is also referred to as a negative regulator of PI3K signaling [49].

The PI3K signaling pathway is one of the possible targets for overcoming breast cancer therapy resistance [50]. Aberrations in this pathway are frequently detected in breast cancer; as a result, tumor cells grow irregularly and become resistant to endocrine therapy [51]. The mutation in *PIK3CA*, which encodes the p110α catalytic subunit of the PI3K, is the most prevalent in HR+ breast cancer. Mutations in *PIK3R1*, which encodes the p85α regulatory subunit of the PI3K, have also been identified in HR+ breast cancer [52]. In a study [53] on 458 samples of breast cancer, 151 samples had *PIK3CA* mutations (33.0%), 10 samples had *PIK3R1* mutations (2.2%), and 283 samples had *PIK3R1* underexpression (61.8%).

*PI3KR1* acts as a tumor suppressor [40] and encodes the p85α regulatory subunit, which inhibits the kinase activity of p110α. Additionally, p85α participates in the stabilization and localization of p110-PI3K activity [54]. Its primary function is to bind to, stabilize, and inhibit the p110 catalytic subunit until RTK is activated [55]. In other words, p85 inhibits the function of p110 during arrest conditions. When p85 at the SH2 domain becomes phosphorylated, the PI3K function as a kinase is activated. When RTK is activated, it needs the p85α regulatory subunit to activate class IA PI3K. The interaction of p85α with activated RTK or adaptor proteins, such as insulin receptor substrate-1 (IRS1) and IRS2, alleviates the basal repression of p110; consequently, the catalytic subunit also becomes activated [56]. The motif is a specific binding site for the SH2 domain of p85α, which must be bound to activate the p110 catalytic subunit of PI3K [57]. The underexpression or genetic alteration of *PIK3R1* can impair the function of the p85α regulatory subunit, resulting in an increased PI3K pathway activity [41, 42]. Furthermore, oncogenic mutations in the PI3K enzyme can affect the kinase domain, but they impede the ability of p85α to inhibit p110, resulting in an unchecked constitutive activity [58]. This finding was also supported by a previous research [40], which demonstrated that the increased PI3K activity due to the loss of the p85α function can result in EGF-independent cell growth. This factor contributes to tamoxifen resistance. Additionally, cancer cells lacking the p85α function became more sensitive to EGF than cells with a normal p85α function. Thus, cancer cells have irregular growth [40].

Numerous studies have demonstrated that OA can inhibit the PI3K signaling pathway. Li et al. [59] administered OA to prostate cancer cell lines and found that it decreases PI3K and p-AktSer-473 phosphorylation while maintaining a constant total amount of AKT. Yang et al. [43] suggested that administering OA alone, a PI3K inhibitor alone, or both simultaneously to a mouse model of renal ischemia–reperfusion injury can significantly decrease the levels of p-AKT/Akt, PI3K, and PDK1. Additionally, the OA pretreatment of mice during the acute phase of hepatic ischemia–reperfusion increases the phosphorylation of p-PI3K (p85α) and p-AKTSer-473 and decreases the total amount of AKT [60]. However, [61] demonstrated that the administration of ursolic acid, an isomer of OA, decreases the amount of p-AKT. This finding suggests that the decreased PI3K/p85α expression may result in decreased AKT phosphorylation, thereby lowering the PI3K/AKT activity of the signaling pathway. Wu et al. [62] conducted another experiment by treating the MCF-7 cell line with SZC015, a synthetic OA derivative compound. The administration of this compound simultaneously downregulates the p110α catalytic and p85α regulatory subunits. Interestingly, the p-AKT/AKT ratio increases, indicating that p-AKT phosphorylation increases even though the downstream signaling pathway has been eventually downregulated [62]. These studies have revealed that OA can inhibit the downstream activity of the PI3K signaling pathway. Molecular docking also confirms that OA can inhibit p85 activation even though the number of amino acids involved is lessened. However, its potential should be validated via in vitro tests. Inhibiting p85α, the non-catalytic subunit of phosphatidylinositol 3-kinase, has a strong antitumor effect on human breast cancer cells [63].

A recently published review article discussed the development of OA and its derivate for anticancer therapy via several mechanism, including induction of apoptosis, regulation of cell cycle, inhibition of angiogenesis, inhibition of stem cells, reversal multidrug resistance, and increase immune system [64]. OA belongs to the biopharmaceutics classification system (BCS) IV, which has poor water solubility and permeability and is metabolized by cytochrome P450 such as CYP3A, which will further reduce its bioavailability [65]. Modification of the molecular structure of OA may increase the potency of this compound. An OA derivate namely CDDO-Me was found to inhibit PI3K/AKT/MTOR signaling in pancreatic cancer cells [66]. In addition, their bioavailability needs to be considered for the development of OA for preclinical and clinical studies. Making the right formula and the proper administration is also a challenge in developing OA for overcoming breast cancer resistance to tamoxifen. This research was conducted to help develop a therapy for overcoming tamoxifen resistance. Therefore, the findings of studies supporting the role of OA in overcoming tamoxifen resistance should be confirmed by further research.

## Conclusion

OA likely targets *PIK3R1* to overcome tamoxifen resistance in breast cancer therapy. Molecular docking shows that OA can inhibit p85 activation. OA acts molecularly by inhibiting p85 activation, leading to the inhibition of the downstream activity of the PI3K signaling pathway, causing breast cancer patients to respond to tamoxifen therapy once again. However, these findings should be confirmed through further research, including specific in vitro and in vivo studies.

## Supplementary Information

**Additional file 1: Supplementary Figure 1**. Distribution of the value obtained from the GEO database. The distribution of data for data series GSE67916 (A) and GSE86871 (B) is quite good.

**Additional file 2: Supplementary Table 1**. DEGs of tamoxifen-resistant MCF-7 cell line (GSE67916). **Supplementary Table 2**. DEGs of the MCF-7 cell line treated with OA (GSE85871). **Supplementary Table 3**. GO enrichment analysis of the OTGs : Biological Process. **Supplementary Table 4**. GO enrichment analysis of the OTGs : Cellular component. **Supplementary Table 5**. GO enrichment analysis of the OTGs : Molecular Function. **Supplementary Table 6**. KEGG pathway enrichment analysis of the OTGs.

## Data Availability

All data generated or analyzed during this study are included in this published article [and its supplementary information files].

## Abbreviations

DAVID: Database for Annotation, Visualization, and Integrated Discovery
DEGs: Differentially expressed genes
ER: Estrogen receptor
GEO: Gene Expression Omnibus
GO: Gene ontology
HR: Hormone Receptor
INSERM: Institut National de la Santé et de la Recherche Médicale
KEGG: Kyoto Encyclopedia of Genes and Genomes
MCC: Maximal Clique Centrality
mTOR: Mammalian target of rapamycin
OA: Oleanolic acid
OTGs: Potential oleanolic acid target genes
PDK1: Phosphoinositide-dependent kinase 1
PIP2: Phosphatidylinositol 4,5-bisphosphate
PIP3: Phosphatidylinositol 3,4,5-triphosphate
PTEN: Phosphatase and Tensin
PTGs: Potential target genes
