# Supplementary figures and images for: Functional network analysis of p85 and PI3K as potential gene targets and mechanism of oleanolic acid in overcoming breast cancer resistance to tamoxifen
Source: J Genet Eng Biotechnol. 2022 Apr 28;20:66. doi: 10.1186/s43141-022-00341-4 (PMC9050990; doi:10.1186/s43141-022-00341-4)

## Slide 1
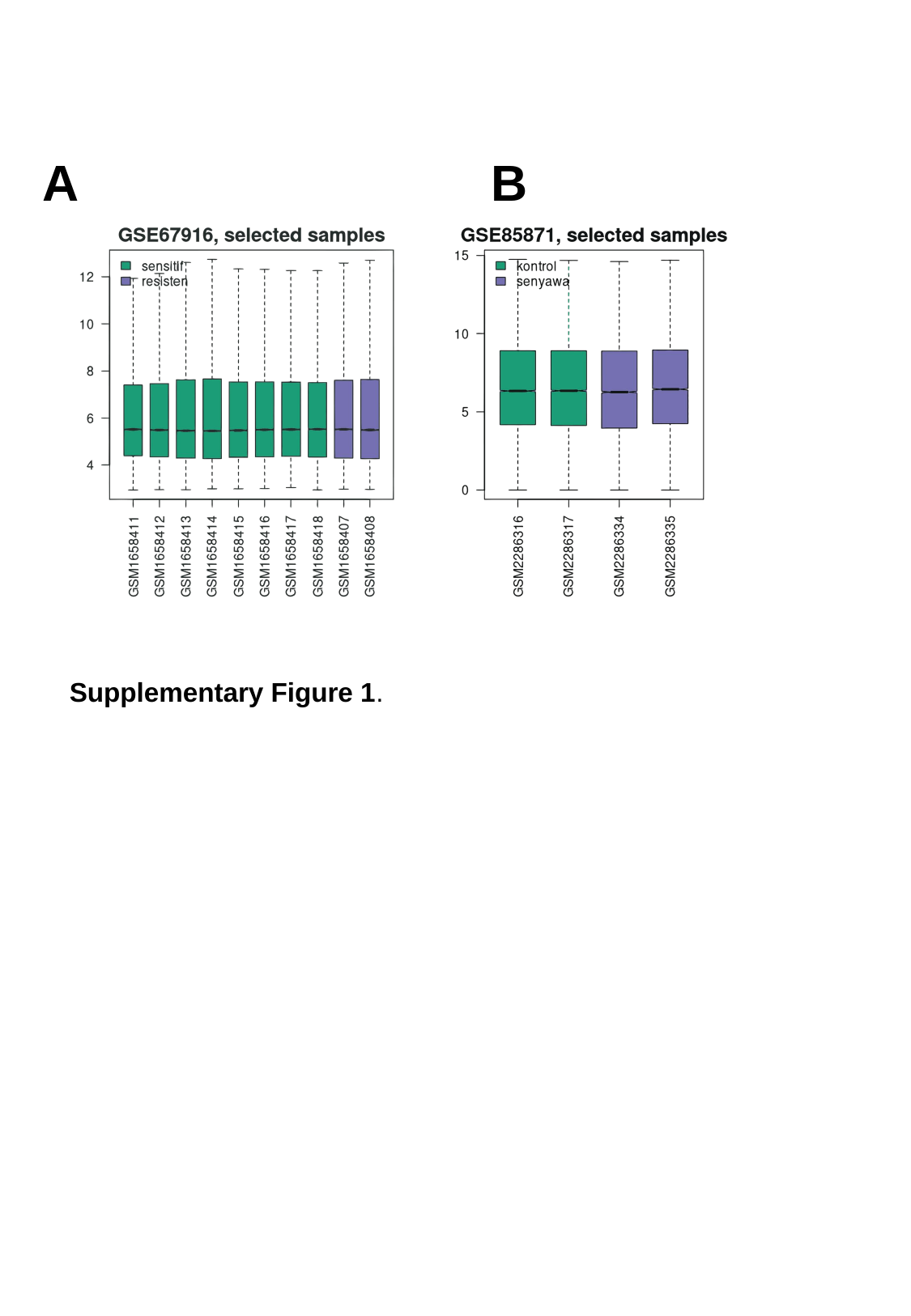

A
B
Supplementary Figure 1.

Supplement: Supplementary file 1 — Additional file 1: Supplementary Figure 1. Distribution of the value obtained from the GEO database. The distribution of data for data series GSE67916 (A) and GSE86871 (B) is quite good. [file 43141_2022_341_MOESM1_ESM.pptx]
